# Commentary: Blurred lines: Performance Enhancement, Common Mental Disorders and Referral in the U.K. Athletic Population

**DOI:** 10.3389/fpsyg.2016.01709

**Published:** 2016-11-03

**Authors:** Martin Eubank

**Affiliations:** School of Sport and Exercise Science, Liverpool John Moores UniversityLiverpool, UK

**Keywords:** common mental disorders, sport psychologist, competence, philosophy, referral

Given the personal and sensitive nature of mental disorders, and the stigmas to disclosure that often exist in high performance sport, it was not surprising to learn from Roberts et al. (2016) that the Sport Psychologist, with whom the CMD athlete is likely to have a confidential, trusting, and empathic working relationship, is the person they are most likely to seek out for support. For me, the capacity of the Sport Psychologist to provide the support being sought depends on their competence to both consult with, *and* refer, athletes with CMD, and the extent to which their own practice philosophy and counseling based training informs their consultancy approach. These two issues form the focus of this commentary.

## Common mental disorders and practitioner competence: issues of consultation and referral

Roberts et al. (2016) provide effective case study examples of the blurred lines that exist within the referral process. For me, this implicates practitioner competence, which, as an ethical principle, includes the “ability to function optimally within the recognized limits of knowledge, skill, training, education, and experience” (British Psychological Society, 2009, p. 15). For CMD, consultancy competence across the psychotherapy, counseling, and mental skills training continuum is no bad thing! In being able to use the counseling “middle ground,” the Sport Psychologist is fulfilling an essential support need for their client through their capacity to listen and be empathic in an unconditional and non-judgment way.

That said, this does not mean they are acting as competent Clinical Psychologists who can solve clinical problems. The difficulties associated with athletes being referred to “unknown” clinical psychologists with whom they have no relationship is a difficult challenge to overcome, however good the clinician might be at their job. Recently, a “system approach” to supporting high performance athletes with CMD has received attention in the literature (Bickley et al., 2016; Rotheram et al., 2016). For me, this represents an interesting model of practice that is worthy of further consideration in the treatment of CMD in athletes. The approach involves an *integrated* clinical and sport psychology method, where the clinical psychologist works collaboratively alongside the sport psychologist, and the wider science and medicine team and coaches, to derive an agreed case formulation of the problem and the approach to be used, which is then implemented by the Sport Psychologist under close clinical supervision and governance. This close collaboration continues in the monitoring undertaken to evaluate the ongoing effectiveness of the intervention. While this may not be without its contention, the reality of performance sport can sometimes mean that full clinical psychology referral is simply not available, so the decision about how to proceed can often come down to whether clinically supervised support vs. no support is the best option. This collaborative systems approach emphasizes the importance of following the ethical decision-making process afforded by the code of ethics and conduct to effectively identify acceptable boundaries of competence, and also enables the existing strong and functional client–practitioner relationship to be used to facilitate the intervention. The approach would appear to offer a viable referral approach for CMD and other psychological disorders in sport, perhaps make the lines slightly less blurred.

## Common mental disorders and practitioner philosophy: performance and care in equal measure

Practitioner philosophy is defined as “the consultant's beliefs and values concerning the nature of reality, the place of sport in human life, the basic nature of a human being, the nature of human behavior change, and also the consultant's beliefs and values concerning his or her potential role in, and the theoretical and practical means of, influencing their clients toward mutually set intervention goals” (Poczwardowski et al., 2004, p. 449). Eubank and Hudson (2013) observed that it is common for Sport Psychology trainees to be uncertain about the practitioner philosophy that underpins their applied work, and is often the aspect of professional practice that neophyte practitioners find most challenging to embrace, document and articulate (Eubank, 2016). Development of a congruent philosophy that underpins the quality of support service delivery requires an understanding of one's own beliefs and values and their translation to “self in practice” Having concern for the growth and development of people (athletes), including the vulnerable (someone with CMD), for example, represents a core belief and value common to most practitioner psychologists.

Roberts et al. (2016) identify a critical contextual reality that is worthy of further commentary. Practitioner Psychologists work in a context, not a vacuum. Thus, congruent philosophy is about self, self in practice, but also *self in the practice context*. High performance sport environments are laden with intense, uncertain and challenging realities built around a constant pre-occupation with achievement, outcome and success. The importance of “getting the culture before it gets you” (Eubank et al., 2014) is a key survival challenge. As Roberts et al. have argued, it is the nature of this environment that can, in some cases, fuel the onset of CMD, and also the unfortunate likelihood that some athletes will want to conceal them from view for fear of being perceived as less “mentally equipped” to make a positive contribution to performance driven objectives. This is unhealthy.

Support for CMD in sport arguably requires a “person first, athlete second” practitioner philosophy and a broad and holistic model of approach that accommodates athlete well-being. However, the high performance culture of sport means that the Sport Psychologist's own survival strategy for effective and sustained working practice, and their job, places an almost inevitable emphasis on performance enhancement (see Brady and Maynard, 2010). Sport Psychologists who philosophize their practice based on client welfare as the primary concern often encounter dissonant moments between the needs of the client as they perceive them, and the demands of the organization in which they operate. To help with this, an approach to sport psychology support that is personally congruent but also environmentally/culturally resonant is advocated, and there is value in using both focused/narrower interventions and broader counseling-based approaches to support “performance and care” agendas in high performance sport in equal measure (Eubank et al., 2014).

## Author contributions

The author confirms being the sole contributor of this work and approved it for publication.

### Conflict of interest statement

The author declares that the research was conducted in the absence of any commercial or financial relationships that could be construed as a potential conflict of interest.
